# Experience rates of current and future geothermal power technologies

**DOI:** 10.1016/j.isci.2026.117113

**Published:** 2026-08-12

**Authors:** Florian Mueller, Bjarne Steffen, Tobias S. Schmidt

**Affiliations:** 1 Energy and Technology Policy Group, ETH Zurich, Zürich, Switzerland; 2 Einstein School of Public Policy, ETH Zurich, Zürich, Switzerland

**Keywords:** energy innovation, base load, learning curve, learning rate, geothermal, expert elicitation

## Abstract

Geothermal power generation could play an important role in future energy systems, provided it experiences strong cost reductions. However, unlike for other renewables, data on geothermal cost dynamics are sparse. We address this gap by estimating global and local (country-level) experience rates using a newly compiled dataset of all 514 plants built over seventy years, 206 of which have cost data. We estimate a negative global experience rate (−20%) with 97% confidence for hydrothermal systems, indicating rising global costs. On the country level, we observe both negative and positive experience rates. Explaining these patterns, based on 51 expert interviews, we identify six factors, including varying hydrogeology, regulatory density and variance, and knowledge spillovers. We further assess two emerging technologies: enhanced and closed-loop geothermal systems. While they may exhibit higher local experience rates than hydrothermal in suitable contexts, they are unlikely to exhibit rapid global cost declines similar to other renewables.

## Highlights

• New dataset covers all 514 geothermal plants built over the last seven decades
• Global geothermal costs have risen with deployment (experience rate of −20%)
• Local experience rates range from +18% in the United States to −53% in New Zealand
• Enhanced and closed-loop systems unlikely to change cost dynamics

## Graphical abstract

**Figure:**
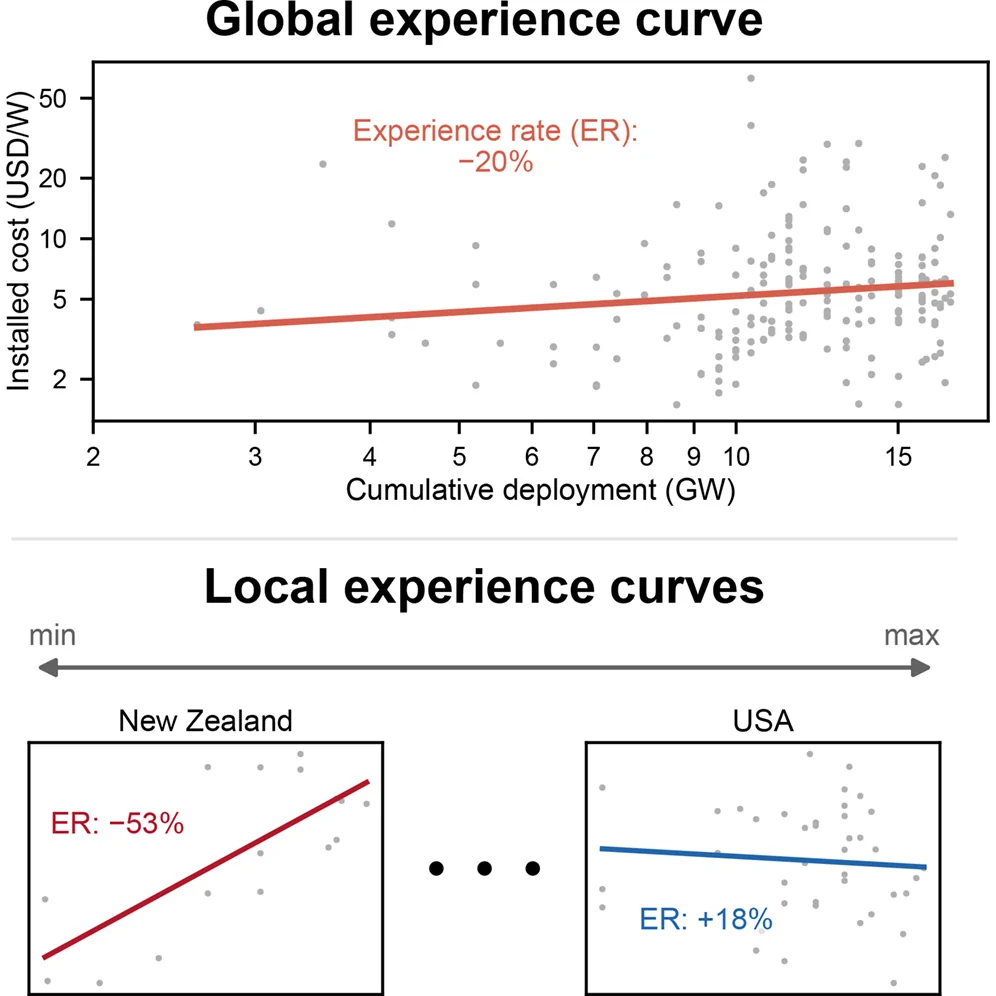

## Teaser

Energy policy; Energy sustainability; Energy systems

## Introduction

Wind and solar photovoltaics (PV) have experienced dramatic cost reductions and are poised to play a central role in future low-carbon electricity systems.^1^ Both technologies produce fluctuating renewable energy, requiring complementary storage technologies^2^ but also “firm” clean energy sources which provide flexible and controllable supply.^3^ One such technology is geothermal power, which utilizes subsurface heat and offers renewable firm electricity on a global scale.^4^^,^^5^ Despite its long-standing presence, geothermal power deployment has been slow, and its application remains limited to a few, mostly volcanic, regions.^4^ One reason is the high cost of drilling in regions where the heat source sits deeply underground.^4^^,^^5^^,^^6^^,^^7^

Recently, interest in geothermal power has risen sharply, driven by great hopes that its costs will fall substantially.^4^^,^^8^ However, while multiple studies on the cost of geothermal power exist,^9^^,^^10^^,^^11^^,^^12^ their cost *dynamics* remain poorly understood.^8^ Reasons for this are the scarcity and quality of long-term data and local heterogeneity of the technology and its use environment, and are discussed in more detail in the following paragraph. This lack of understanding is in sharp contrast to other renewable energy technologies, where cost dynamics have been analyzed in depth and found to be a decisive factor in their competitiveness vis-à-vis each other but also fossil fuel-based technologies.^13^^,^^14^^,^^15^ An important and well-established concept in this regard is the experience curve (often also called learning curve).^14^^,^^16^^,^^17^ It describes the empirical phenomenon that the relative cost of a technology is reduced by a relatively constant percentage—the so-called experience rate (ER)—per doubling of cumulatively installed capacity.^13^ Experience curves are arguably the most reliable method to assess technological cost trajectories.^18^ The two existing global studies on geothermal experience curves report strongly negative ERs, between negative 29% and negative 100%.^19^^,^^20^ These numbers stand in sharp contrast to the International Energy Agency’s 2024 geothermal report, which uses expert elicitation to project cost decreases of up to 80% by 2035,^4^ implying extremely high ERs even under optimistic deployment scenarios.

The existing analyses, of which we provide an overview in Table S1, are plagued by several issues related to data: (1) There are too little data to construct reliable experience curves. The literature recommends considering data over at least three doublings of capacity,^21^ but the dataset from IRENA that many authors rely on only covers 0.3 doublings of capacity. Also, its data only starts from 2008, providing little insight into true long-term trends. (2) The data used lacks proper verification, and our analysis has found several flaws in the data. This leads to incorrect results, and single errors have a major impact due to the small sample size. More generally, the lack of high-quality data has been identified as a major challenge in constructing geothermal experience curves.^13^^,^^22^ (3) The analyses ignore local differences in the cost dynamics. Geothermal power can be expected to have locally differing costs due to a high local cost share and differences in plant design. Ignoring these differences can skew results. For example, the analyses might interpret a shift to higher-cost countries with negative learning. The analyses provide little explanation for the observed experience rates. Without a differentiated analysis of ER drivers, it is hard to reason if and how new technologies or policies can change the past ER.^16^^,^^17^

The hopes for cost improvements are partly based on two emerging geothermal technologies: enhanced geothermal systems (EGS) and closed-loop geothermal systems (CLGS), sometimes also called advanced geothermal systems. These technologies promise greater scalability than the existing hydrothermal technology. Hydrothermal systems rely on rock permeability and the existence of water in the underground, both of which can vary strongly locally and are costly to assess.^23^ EGS uses recently developed fracking technology to make plants independent from natural—often too low—permeability.^24^ CLGS utilize a closed underground heat exchanger with many thin wells and circulating a selected fluid.^25^^,^^26^^,^^27^ Despite the apparent advantages of EGS and CLGS, it remains unclear whether these technologies’ characteristics truly result in substantially lower long-term cost. Ultimately, geothermal power needs realistic long-term improvement in technological learning to become competitive globally.

In this study, we address the uncertainty around geothermal cost dynamics by providing the first robust empirical estimate of global ERs, drawing on a newly compiled dataset covering all 514 geothermal power plants that have been commissioned over the last seven decades. To understand the large variation in reported costs across geographies, we also estimate local, i.e., country-specific ERs. Because experience curves quantify the rate of cost change but not its underlying causes, we complement this quantitative analysis with 51 interviews with geothermal experts across the world, identifying key drivers of and barriers to cost reductions. This mixed-methods design, established in prior learning studies of solar PV,^28^^,^^29^ wind,^29^ nuclear power,^30^ and financing cost of renewable energy in general,^31^ lets us link the observed ER patterns to their structural determinants. Finally, we address the future technologies EGS and CLGS. Drawing on a recently established framework of technology characteristics and expert ratings,^32^^,^^33^^,^^34^ we assess the potential for these emerging technologies to enable faster and global cost declines. Together, these steps provide a comprehensive understanding of past and future geothermal cost dynamics and their implications for energy policy and modeling. Our contribution is primarily empirical: We provide the first consistent global learning rate estimate for geothermal power based on a comprehensive, multi-decade dataset and offer directly actionable insights for energy system modelers seeking to understand where and under what conditions geothermal can become cost-competitive.

## Results

### Deployment of geothermal power globally

**Figure 1:**
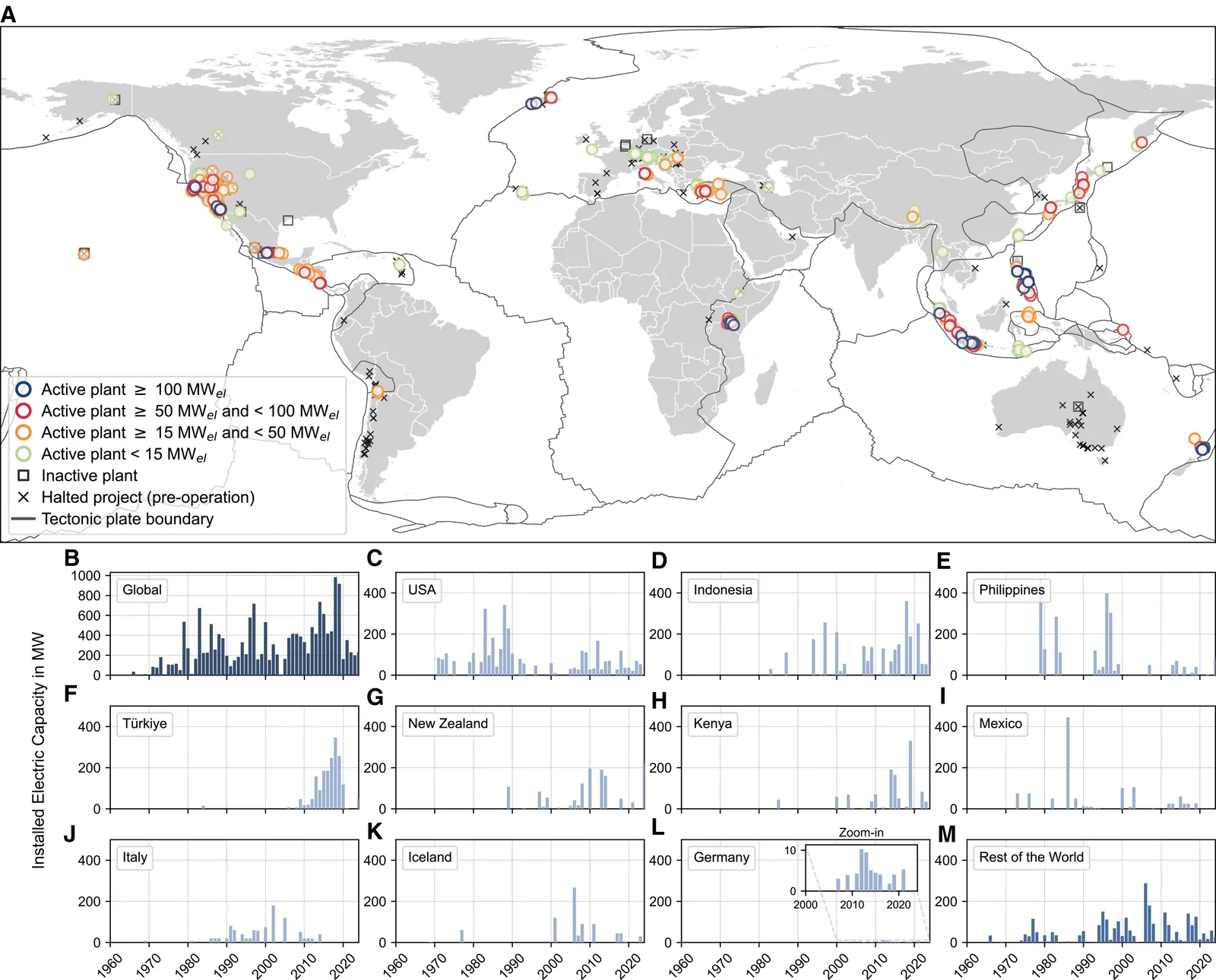
Global distribution map and historical deployment of geothermal power plants Map of geothermal power plants (A), historical deployment globally (B), in the top ten countries by cumulative deployment (C–L), and in all remaining countries combined (M). See Figure S1 for cumulative deployment.

Geothermal power has been used since the early 1900s.^35^ As of mid-2025, projects exist in more than 60 countries (Figure 1A), yet geothermal accounts for only 0.5% of global renewable electricity generation.^4^ Recent growth has been modest, with just 0.2 GW of new capacity added in 2024 (Figure 1B), compared to 114 GW in wind turbines and 452 GW in solar PV.^36^

Many large plants are in geologically highly active regions, especially around the Pacific Ring of Fire (Figure 1A). Multiple halted projects (pre-operation) illustrate the development challenges associated with geothermal deployment beyond the most advantageous regions.

Global deployment varies strongly over the years (Figure 1B) as few large plants in geologically highly active regions dominate capacity additions. While deployment generally increased over the last decades, there has been a downward trend in capacity additions since a peak in 2019, when the newly installed capacity was close to 1 GW. At the national level, deployment is even more irregular (Figures 1C–1M).

Examples illustrate the variations in deployment. In the United States, capacity additions peaked in the 1980s, when multiple big fields strongly expanded production (Figure 1C). Recent capacity additions are lower, as they mostly focus on the edges of existing fields, less attractive new fields, and the repowering of old, partly depleted fields. Türkiye experienced a large geothermal boom in the 2010s, boosted by subsidies. After subsidies were phased out, deployment plummeted (Figure 1F).^37^ In other countries, deployment patterns were mostly driven by site availability and policy changes as well (Note S1).

### Estimating and explaining global experience rates

**Figure 2:**
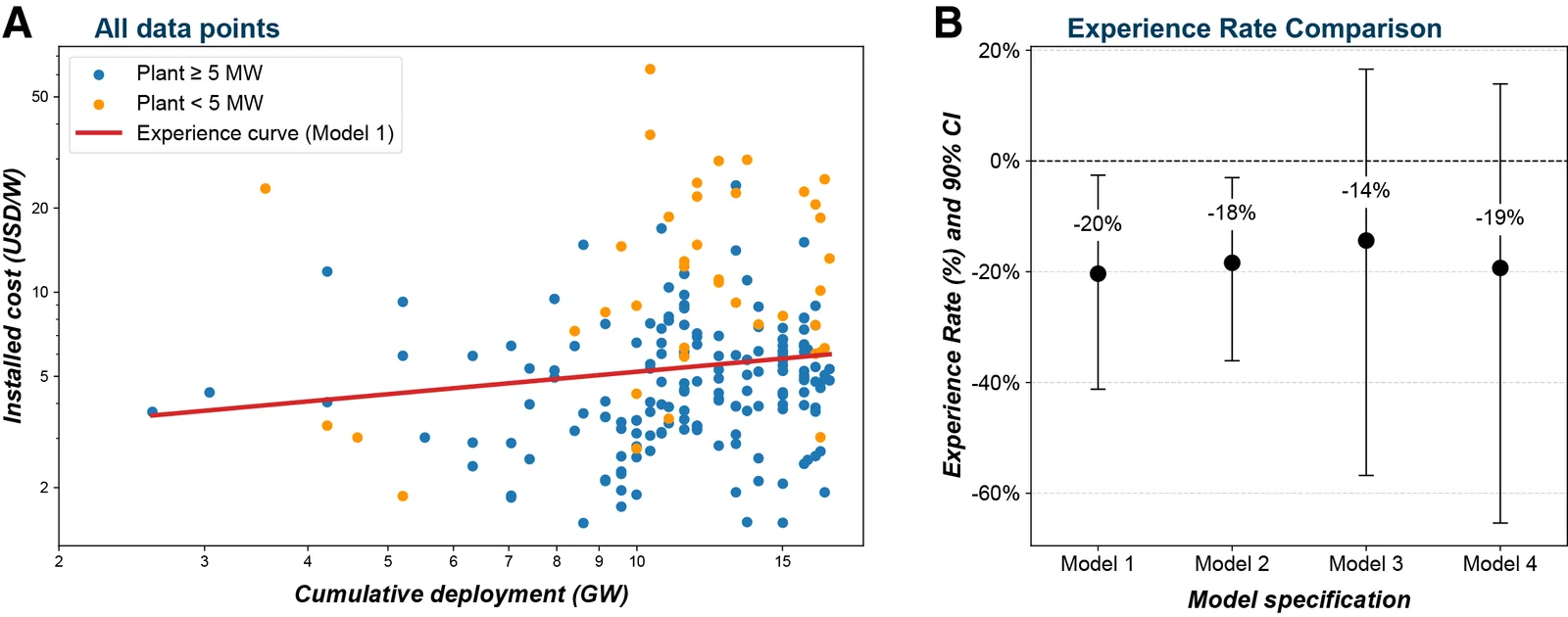
Plant costs and global experience curves of geothermal power Plant costs in 2024 USD and global experience curve of Model 1 (A) and ERs with alternative models (B). Model 1 regresses over all plants. Model 2 includes only plants with capacities of at least 5 MW. Models 3 and 4 mirror Models 1 and 2, respectively, but adjust for exchange rate distortions. Detailed parameters for all models can be found in Table S3. In (A), global cumulative capacity per plant is assigned per the capacity at the beginning of the year in which the plant became operational. In this, no mutual learning is assumed between plants becoming operational within the same year and thus constructed in parallel.

Figure 2A displays all 206 geothermal plants with cost data, plotted against cumulative deployment, which is based on all 514 projects (see STAR Methods for details). We estimate a global ER of −20% [90% confidence interval (CI): −41.2% to −2.6%] (Model 1 in Figure 2B). The wide CIs stem from the fact that we use all projects with all their site-specific conditions. Notably, many early plants had low installed costs around 3 USD_2024_/W, whereas several recent plants exhibit significantly higher costs, some exceeding 10 USD_2024_/W. When we exclude small plants (i.e., the 16% of the sample with an electrical capacity below 5 MW), the estimated ER is not much affected, however (Model 2).

In estimating global ER, adjusting for exchange rate fluctuations can help to avoid biased ER estimates (see STAR Methods).^38^ The exchange rate-adjusted ER in Model 3 (−14%) is slightly higher than in Model 1 as the effect of US-Dollar appreciation against softer currencies is accounted for. The currency depreciation effect mostly affects small plants, which is why Model 4 shows less sensitivity to currency corrections and its ER (−19%) is close to Model 2. Models 3 and 4 have wider CIs than Models 1 and 2 due to the larger impact of some soft currencies’ fluctuations against the US Dollar.

Observers might note some statistical peculiarities in our data and results. First, there is a large spread between the cheapest and most expensive projects. We explain more about the techno-economic reasons for these variations below and note that cost variations of about one order of magnitude in a single year are the norm in all large-scale renewable energy technologies.^36^ Second, as a consequence of these fluctuations and the fact that we use plant-level data, the observed confidence intervals are wider than in most studies that use yearly averages. Third, the wide confidence intervals in some models cross the zero-line, facilitated by ERs that are closer to zero than in some other technologies. In the literature, there are many technologies without a CI clearly in the positive or negative range. Depending on the study and model, these include offshore wind, onshore wind, hydropower, nuclear power, concentrating solar power, bioenergy, and coal and gas generation.^19^^,^^20^ Many other studies do not include confidence intervals, but aggregate over different time intervals or regions and also show both positive and negative ERs for many technologies.^13^^,^^21^ Only solar PV has its CI entirely in the positive range in all studies. This is due to solar PV’s generally high ER, e.g., +34% according to Yao et al. (2021), and its globally standardized nature, which limits cost fluctuations.

While the experience curves quantify how geothermal costs have evolved, they cannot reveal *why* learning has stalled globally and why it varies so strongly across countries. The drivers behind these patterns are not contained in the cost data itself but are distributed among the practitioners who develop the plants. We therefore draw on 51 expert interviews. Their value lies less in uncovering individual barriers that might already be familiar with geothermal specialists, but rather in making them analytically useful: We separate drivers that systematically and globally suppress learning from those that act only locally, at specific times, or for specific technologies; we group them into broader factors; and we phrase these factors so they transfer to other technologies and to the wider scholarship on technological learning. This yields six factors that explain the observed global cost dynamics (Table 1). These relate to different ER determinants described in the literature.

**Table 1:** Factors explaining geothermal-specific learning dynamics and effect on learning dynamics

Factor	Explanation	Interview quote	Effect
**Diminishing productivity of new sites**	Easily accessible high-quality resources with high temperature gradients are rare. Thus, project developers must successively turn to less productive or more costly sites	“The best resources have been captured early. [Today,] we do much more well redrilling and plants at the edges of existing fields [where yield is less and more uncertain]. Also, the wells become more complex.” (Interview 9)	Increases cost of later plants
Related determinant:			
Resource effect			
**Project design complexity**	Degree of interdependence between project phases and actors. Higher complexity increases project risks and coordination needs and slows learning-by-doing	“[Projects are] complex, with many specialists needed. […] and, we need many iterations.” (Interview 7)	Slows learning due to need for many interactions between actors
Related determinant:			
Design complexity			
**Varying hydro-geology**	Hydrogeology can strongly vary between sites, thus requiring re-assessments of geology and different knowledge to drill and to operate reservoirs	“[There are] very few economies of scale […]. One can learn very little from other geothermal projects — unless they are right on your doorstep.” (Interview 7)	Slows learning due to customization and lower scaling of the exact same setup (no repetition)
Related determinant:			
Need for customization			
**Tacit nature of drilling knowledge**	Much of the knowledge required for projects is tacit. This knowledge resides in drilling teams and project managers and is hard to transfer	“[It takes] two years for a drilling crew to become fully working. […] any successful project requires experts who understand the local geology” (Interview 39)	Constrains knowledge to local sites and teams
Related determinant:			
Knowledge transfer			
**Regulatory density and variance**	Regulation varies significantly in place and time. Reasons for this lie in differing seismic risks, vulnerability of water reserves, and population density. Regulatory density has increased	“Regulations, permitting and subsidy are more complicated than geology. […] There are differences on a communal level […] and there are new surprises every day.” (Interview 7)	Increases cost and slows learning due to increased project design complexity and customization
Related determinant:			
Need for customization			
**Knowledge transfer from oil and gas industry**	Existing oil and gas knowledge positively impacts geothermal projects. This includes drilling know-how, equipment and personnel availability, familiarity of regulators with drilling, public acceptance, and data availability concerning the local geology	“[In our region] there used to be good knowledge, but it was lost with [oil and gas firm name] that disintegrated. They were the last ones.” (Interview 33)	Decreases cost without a need for geothermal investment
Related determinant:			
Knowledge transfer			

The effects are bold. The factors are categorized along established broader phenomena, which are discussed in more depth later in the paper. More interview quotes can be found in Table S5.

*Diminishing productivity of new sites* can drive up costs from project to project, as e.g., surface-near heat reservoirs are exploited first, thus countering the experience curve effect. In the literature, this factor is described as “resource effect.” A plot of the available resources against their (increasing) cost is known as the resource supply curve, and such curves can be found in the literature.^39^^,^^40^^,^^41^ The most important aspect of site productivity in geothermal is temperature, since higher temperatures have a double-positive effect of increasing energy of the fluid and higher energy extraction efficiency (from Carnot efficiency). We find that resource temperatures are declining globally and in all important countries (Figure S2). Next to this, increasing resource depth also plays a role in increasing project costs, as does the decreasing “drillability” of the rock and the decreasing permeability of the targeted formation. Furthermore, increasing remoteness of sites and decreasing knowledge about underground properties can increase costs of new sites. The diminishing productivity of new sites is also relevant in other renewable energy technologies, such as hydropower, where most suited dam sites are already exploited.^42^ In contrast, resource depletion plays only a minor role in wind power, where wind-rich sites remain widely available, and is barely relevant in solar PV.^7^^,^^43^^,^^44^

*Project design complexity* hinders fast improvements and learning-by-doing due to long improvement cycles within projects. The iterative nature of projects with many actors increases coordination cost, the risk of bottlenecks, and the cost of experimentation, as interviewees pointed out. In the literature, high design complexity is named as a reason for the low experience rates of technologies such as nuclear or carbon capture and storage plants.^30^ In contrast, the project design complexity of solar PV plants is much lower and experience rates much higher.^32^

*Varying hydrogeology*, or more broadly, spatial heterogeneity in the physical environment, slows learning according to our interviews. In geothermal, every reservoir differs in terms of rock permeability, fluid chemistry, and thermal gradient, necessitating bespoke solutions. This factor drives the need for customization, which has been associated with lower experience rates. It is also observed in hydropower, where dam design is highly site-specific, and biomass, where the fuel’s physical properties vary across locations.^32^ Wind energy also faces site-specific differences in wind profiles and ground conditions, but standardized turbine classes can accommodate most of this variation.^45^ In solar PV, geography has almost no bearing on core system design, enabling very high standardization and scale economies.

The *tacit nature of drilling knowledge* is largely unique to geothermal and unparalleled in other forms of renewable energy. According to our interviews, much of the expertise resides within localized drilling crews and cannot be easily codified or transferred. This factor sharply contrasts with installation and operational procedures in wind and solar PV, which are much less tacit and well-documented and thus more easily replicated across sites.^21^ Literature has shown that knowledge transfer between actors is highly relevant for learning in renewables.^46^^,^^47^^,^^48^

*Regulatory density and variance* also impede geothermal cost reductions, as many interviewees pointed out. Environmental and permitting requirements vary widely across jurisdictions and change over time, particularly with respect to subsurface risks such as induced seismicity and groundwater protection. Regulatory variance as a driver of the need for customization and thus reduced experience rates has been described in the literature.^32^^,^^49^ However, while regulatory variance exists to some extent for wind power, the implications differ: In wind, compliance typically involves adapting peripheral systems (e.g., siting or noise control), whereas in geothermal, regulation affects core project design and thus has a major impact on cost.

*Knowledge transfer from the oil and gas* industry largely benefits geothermal projects but is also important to other clean energy technologies.^50^ For example, offshore wind benefits from such spillovers in offshore construction techniques and marine project management.^51^ Importantly, our interviewees stress that geothermal power is also negatively affected when the related oil and gas industry declines (locally).^52^^,^^53^

The combination of the mentioned factors explains the negative historical global ERs. The varying hydrogeology and high regulatory density and variance increase the need to customize each project. Increased customization and (project) design complexity have been shown to negatively correlate with ERs.^32^ That said, these constraints primarily affect subsurface activities. Surface plant components, such as turbines and heat exchangers, are less site-specific and can more benefit from global knowledge accumulation and established supply chains. Importantly, the variance in hydrogeology and the regulatory variance should be lower at the local scale. Furthermore, tacit knowledge is more easily transferred at a local level. Together, these factors imply higher ERs at the local level.

### Local learning dynamics

**Figure 3:**
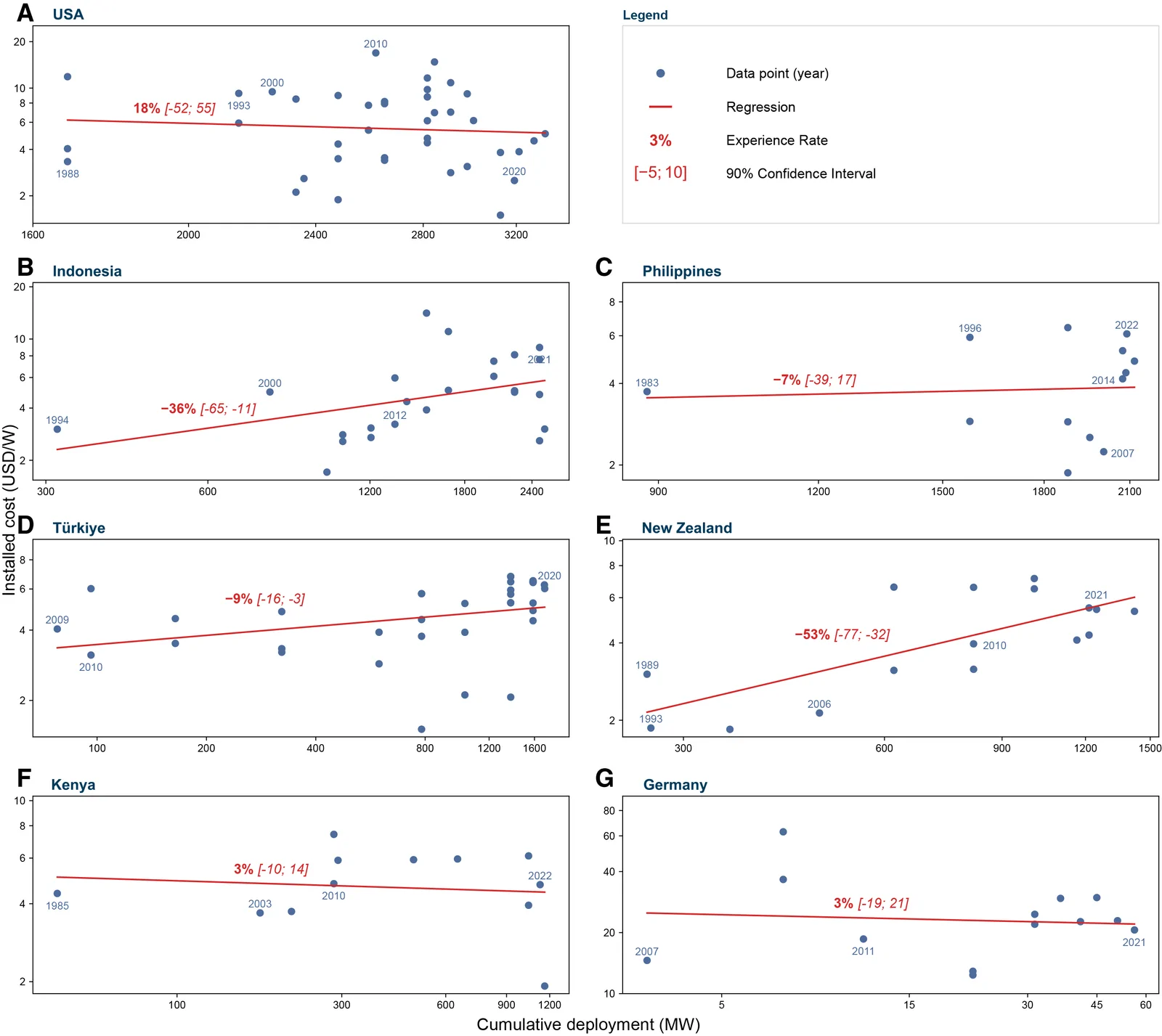
Country-level costs and experience curves of geothermal power (A–G) Country-level experience rates. Costs are in 2024 USD per W; deployment is given in MW. Vertical axis: Plant costs in 2024 USD per W. Horizontal axis: National deployment in MW. Indicative years are shown for the first project per decade in each country. Details about data coverage and model parameters are in Table S4.

Shifting from the global to the national level, we find considerable variation in local ERs. Figures 3A–3G present experience curves for the seven countries with the highest number of plants with available cost data, including the six top countries by cumulative deployment and Germany, which has many small projects. We categorize countries into three groups based on their ERs: the United States with a strong positive ER (+18%), New Zealand and Indonesia with strongly negative ERs (−53% and −36%), and countries with near-zero ERs, concretely the Philippines, Türkiye, Kenya, and Germany. Our interviews help explain the differences in ERs as follows.

In the *United States* (ER: +18%), factors driving up cost or limiting experience effects are less pronounced than in other countries, which explains the high ER: Projects are often located in large and geologically homogeneous structures, limiting the effect of diminishing productivity of new sites and varying hydrogeology. At the same time, regulation is less dense and relatively stable, particularly in sparsely populated desert regions, and many projects are viable without subsidies, limiting exposure to changing support policies in the first place. Additionally, strong cross-industry knowledge transfer from the oil and gas industry supports exploration, drilling, and project management and reduces cost without having to invest to gain these experiences within the geothermal industry. Nevertheless, projects and costs are heterogeneous, explaining the wide CI, even though there is a 70% confidence that the ER is positive (Table S4). *New Zealand*’s negative ER (−53%) is primarily driven by the diminishing productivity of new sites: While early plants were exploiting near-surface reservoirs, newer projects are located in more difficult geological settings, increasing drilling complexity, depth, and thus, costs. Also, in *Indonesia* (ER: −36%), the diminishing productivity of new sites played out as newer projects face worse geological conditions and higher development costs. On top of that, projects moved into more remote, logistically challenging regions requiring additional infrastructure investments. *Türkiye* (ER: −9%) and *Kenya* (ER: +3%) show comparably stable costs due to the high concentration of the projects in place and time. In the *Philippines* (ER: −7%), recent projects fall into two categories: Some in existing fields are cheaper than older ones due to learning effects, while others in new, less explored regions are costlier, resulting in an overall ER near zero. *Germany* shows a positive ER (+3%), primarily due to the now-overcome high costs of early, experimental projects. More recent projects, often in the same reservoirs and under more stable policy conditions, benefited from learning effects, despite challenges from regulatory variance. Nevertheless, absolute costs remain high due to dense regulation and complex project design.

### Learning potential of future geothermal technologies

Our results show that global learning in hydrothermal geothermal power has been limited, with strong variation across countries. This can be explained—among others—by a high need to customize projects and a scarcity of good sites, which implies a rather steep resource supply curve. Emerging technologies raise hopes among industry and investors for faster cost declines as they promise greater standardization^8^ and a flatter resource supply curve, but their limited deployment precludes empirical estimates of ERs. Here, we assess the learning potential of these technologies with an arguably differing maturity using a recently successfully implemented approach: expert ratings of the technologies’ two inherent characteristics (1) need for customization and (2) project design complexity.^32^^,^^33^^,^^34^ Technologies with low customization needs are better suited to replication and scaling. Meanwhile, complexity can slow learning, driven by factors such as tacit knowledge and system integration demands. Specifically, we had experts rate EGS and CLGS’ characteristics along with hydrothermal’s characteristics as a baseline for comparison. A lower complexity and need for customization rating of EGS and CLGS compared to hydrothermal would signify that these technologies can learn faster than hydrothermal, while higher ratings would predict slower learning.

**Figure 4:**
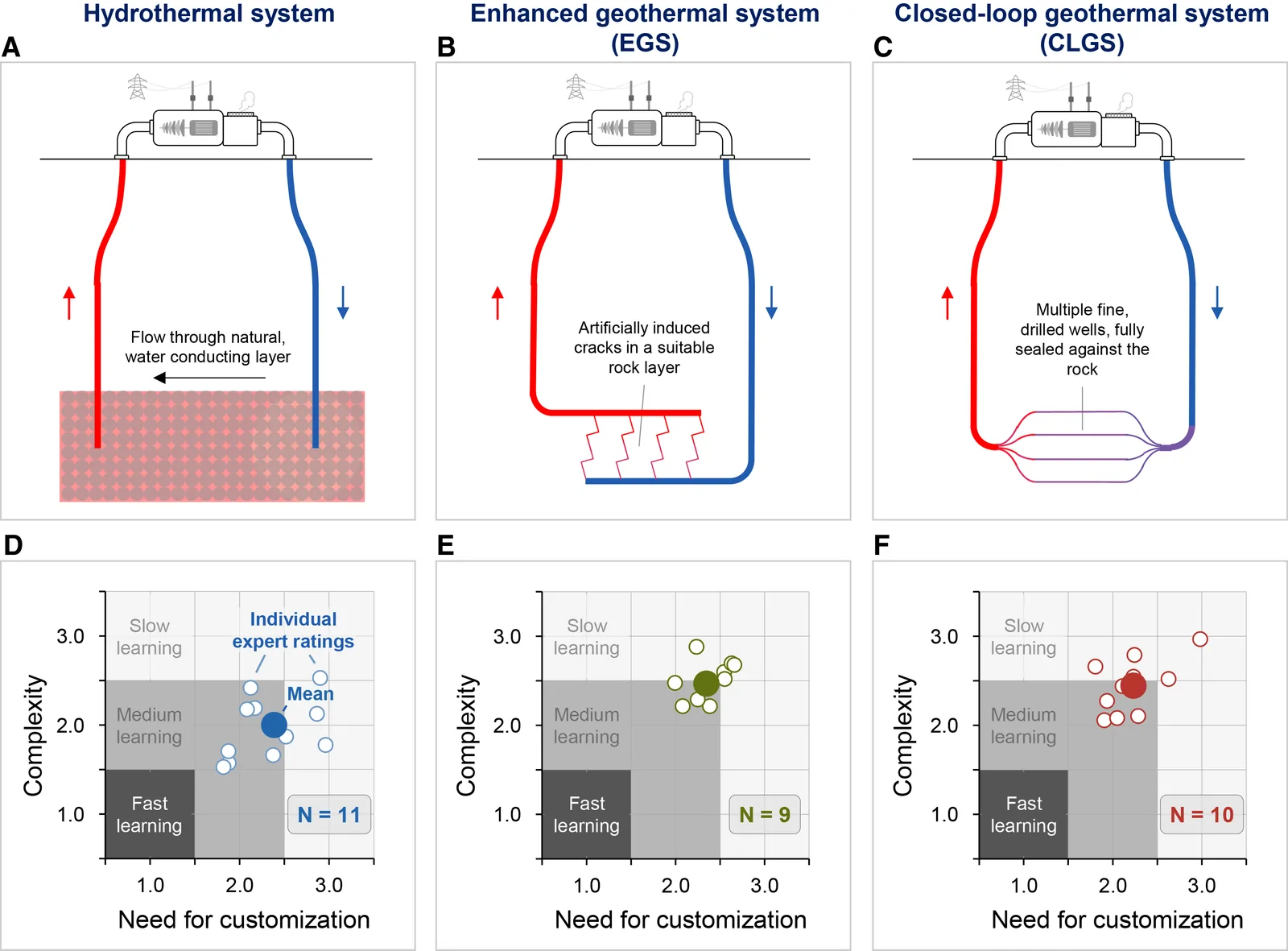
Current and future geothermal technologies and expert assessments of their complexity and need for customization Technical sketches of existing hydrothermal systems (A) and future enhanced (B) and closed-loop systems (C) along expert assessments of complexity and need for customization of the technologies (D–F). N = number of experts that rated each technology. Large circles represent average expert ratings; small circles represent individual ratings. High complexity reflects intricate components and interdependencies, while high customization captures the need to adapt designs to local geological or regulatory conditions. Detailed rating statistics and supporting interview quotes are in Tables S6 and S7, respectively.

For hydrothermal technology (see Figure 4A for a schematic), expert ratings confirm the above qualitative finding that hydrothermal systems are rated as having considerable project design complexity (2.0) and considerable customization needs (2.4), with the greatest variation in ratings among the three technologies (Figure 4D). This heterogeneity reflects the importance of local context in shaping expert perceptions, particularly differences in geology and regulatory density. For emerging geothermal technologies to achieve higher and more consistent ERs, they would need to reduce dependence on local subsurface conditions and regulatory adaptation. Minimizing customization needs, especially in regions with heterogeneous hydrogeology, is a critical prerequisite for scalable cost reductions.

The first emerging technology, EGS, enhances reservoirs by creating artificial fractures using oil and gas industry techniques (Figure 4B). These systems are typically constructed with two or more horizontal wells connected by a network of fracked faults. Experts rated the customization needs of EGS slightly lower than hydrothermal’s (2.4, Figure 4E). Two counteracting dynamics are at play: resource uncertainty is lower, as natural permeability is no longer required, but customization demands rise due to the need to adapt technologies such as fast drilling and fracking to site-specific geology. For instance, achieving competitive drilling speeds depends on optimizing the interaction between drill bit, drilling mud, and operational parameters such as weight on bit, requiring experienced rig crews familiar with local conditions. Experts rated the complexity of EGS higher than hydrothermal systems, at 2.5. In addition to drilling, maintaining adequate long-term permeability adds further technological challenges. Regulatory complexity also increases in densely populated areas, for example, in Central Europe, where fracking-related seismic events have triggered public skepticism and political resistance.^54^

The second technology, CLGS, uses artificially constructed heat exchangers with numerous thin, sealed wells and does not rely on natural fractures or underground water (Figure 4C). As there is no direct contact between the working fluid and the rock, chemical interactions are eliminated. The system relies on an extensive network of narrow wells, increasing total drilling distance by a factor of more than 10 compared to hydrothermal systems (see Note S2).

Experts rated CLGS slightly lower in terms of customization needs (2.2, Figure 4F). This lower value stems from its adaptability to user-specific demands, for example, by adjusting power output or temperature via wellfield design, and from avoiding natural flow dependency. However, advanced drilling technologies still require local adaptation. CLGS’ project design complexity was rated at 2.4, slightly lower than EGS, but higher than hydrothermal. The system’s complexity arises from the need for long horizontal drilling, effective fracture sealing, and highly efficient drilling practices to remain economically viable. That said, CLGS eliminates certain complexities: Sealed systems avoid fluid-rock interactions, and thermosyphon-driven circulation avoids the need for pumps, which is a common reliability burden in hydrothermal systems.^27^

In summary, EGS and CLGS exhibit inherent characteristics that suggest learning may not accelerate drastically on a global scale. Some of the barriers that limited hydrothermal learning can be mitigated. For example, EGS and even more so CLGS reduce reliance on natural water flow and chemistry and local rock properties, which suggests a flatter resource supply curve and faster learning. However, EGS and even more so CLGS come with increased challenges to project design complexity. Thus, EGS and CLGS are unlikely to enable rapid global learning and have experience rates that are still similar to hydrothermal. However, similar to the past learning of hydrothermal technology in the USA, higher local learning is possible, especially in regions with homogeneous hydrogeology and where cross-industry knowledge spillovers can facilitate effective management of unavoidable project complexity.^32^

## Discussion

This study provides the first consistent global and country-level ER estimate and explanation for geothermal power, based on a dataset of 514 plants and 51 expert interviews. Our analysis shows that geothermal power exhibits limited global learning, even though substantial local learning is possible under specific conditions. While global costs have not consistently declined with deployment, several countries, most notably the United States, feature positive ERs due to favorable geological conditions, policy environments, and knowledge transfer from the oil and gas industry. The large variance in local ERs underscores that geothermal learning is highly context-specific, shaped by the interaction of technological, geological, and institutional conditions. As such, geothermal power is unlikely to scale globally in the same way as solar PV or wind, and thus our results cast doubt on the IEA’s 80% cost reduction target by 2035.^4^ Nevertheless, geothermal could become the primary firm renewable power source in specific regional markets where key preconditions are met, such as the United States. There, a recent study about EGS and based on ER assumptions foresees cost reductions around 50%–75% by 2050, down to around 2 USD/W, even when considering the declining availability of resources.^8^ Given that there is sufficient buildout, our empirical results, which showed strong local learning in the United States, support these projections.

These results have implications for multiple audiences, most importantly policymakers, energy system modelers, and scholars of technological learning.

For policymakers, first, geothermal expansion strategies should be grounded in an assessment of regional capabilities and constraints. In areas with favorable hydrogeology—particularly, large and homogenous reservoirs—and an active oil and gas industry, geothermal can scale more easily and cost-effectively. Second, policy can actively improve learning conditions. Besides stable regulation, support for early-stage exploration (e.g., through risk guarantees or public funding), open access to geological and cost data, and incentives for knowledge sharing across projects reduce the need for customization. In addition, partnerships of geothermal actors with the oil and gas industry can strengthen local innovation systems. Examples include facilitating workforce transfer from oil and gas, co-developing modular components, or reusing existing infrastructure where feasible.

These insights extend to future geothermal technologies. While EGS and CLGS are often assumed to improve learning by decoupling performance from subsurface uncertainty,^55^^,^^56^ our results indicate that their potential still depends on managing customization needs and project design complexity. CLGS appears to offer lower customization due to its sealed-loop design and adaptability to user demands, but drilling technology still requires local adjustment. EGS may face continued challenges with fracture stimulation and regulatory approval, similar to those encountered in unconventional oil and gas extraction, which showed high local ERs^57^^,^^58^ and buildout in the US, but did not spread globally. The reasons why unconventional oil and gas extraction could not replicate its strong progress elsewhere parallel those that we find as challenges in EGS: Subsurface heterogeneity preventing fast spillovers, geological conditions unique to the US, especially in resource quality (shale thickness) and ease of accessing it (how easily rock can be fractured), weaker industrial structure outside the US, a lack of strong investors with a high risk appetite outside the US, and increased difficulty of logistics.^59^^,^^60^ Nuclear power offers a second instructive analogy, as it faces the tension between customized and generic design without geological causes. Where reactor programs committed to standardized designs and serial construction by stable supplier and constructor networks, such as France’s fleet buildout in the 1970s and 1980s or South Korea’s standardized reactor series, construction costs remained stable or declined.^61^^,^^62^ Where designs were customized to individual sites, regulatory regimes, and utilities, most prominently in the United States, costs escalated, and learning rates turned negative.^62^^,^^63^ The nuclear case shows that the balance between customization and standardization is not fixed by technology alone but shaped by industrial structure, regulatory stability, and procurement strategy. This suggests that the learning prospects of EGS and CLGS will also depend on institutional choices, not only on their technical characteristics. A third analogy comes from biomass power, where knowledge transfer to new regions is strongly limited by the need to redesign core components for a different, locally produced feedstock.^46^ Future work should investigate how emerging design innovations, such as plasma drilling,^64^ modular surface systems,^30^ or standardized project design,^45^ can help shift geothermal development toward more scalable and replicable trajectories.

From an energy system modeling perspective, our results challenge the default assumption of a single, global cost level and ER across all technologies, as it is currently the standard for integrated assessment models.^65^^,^^66^ For technologies with high customization needs, models ignoring geographic cost heterogeneity and locally confined learning are likely overestimating cost reductions. Accordingly, modelers should consider regionally differentiated experience curves or deploy context-specific cost projections. For geothermal, we advise modelers to consider the factors that drive geothermal learning dynamics from Table 1 for each region separately. Regions with heterogeneous hydrogeology, high regulatory density and variance, and no knowledge transfers from an existing oil and gas industry should be assigned higher ERs than those where the opposite applies. At minimum, this would mean separating the contiguous US from the rest of the world. If a global ER has to be applied, we recommend using a comparably low one for all geothermal technologies.

Finally, for scholars of technological learning, our findings highlight the importance of moving beyond global and paying more attention to local learning dynamics.^67^^,^^68^ Much of the literature has focused on estimating global ERs and decomposing cost reductions into learning-by-doing, R&D, and scale effects. However, the potentially substantive variance in learning across countries remains underexplored^69^—at least for technologies with a high need for customization.^32^ Our study contributes to addressing this gap by linking observed cost trajectories to underlying factors through a mixed-methods research design. We show that diminishing productivity of new sites, varying hydrogeology, regulatory density and variance, and tacit knowledge are not simply context-specific challenges. Instead, they systematically influence a technology’s capacity to accumulate spatially transferable experience. Recognizing the structural basis for these differences helps explain why learning is not globally uniform.

Future work with an even more detailed geothermal dataset could include more granular estimations of ERs, e.g., with a division into geological basins. Furthermore, an enhanced database could allow deriving multi-component ERs and multi-factor ERs, the latter of which could include spillover effects from the oil and gas industry. If carried out successfully, these experiments could even further inform modeling of highly customized technologies.

### Limitations of the study

Our data only present a subsample of all geothermal plants, and the completeness of data varies strongly between countries and decreases for older projects. Despite this, our dataset is still substantially more complete than previous ones. For future technologies, there are more factors determining experience rates apart from complexity and the need for customization, even if these two factors are the most important ones. In addition, different forms of spillovers from other sectors are conceivable; however, these are hard to model, and our approach constitutes a more accurate way of forecasting technology cost than expert elicitation.^18^

## Resource availability

### Lead contact

Inquiries regarding the data and method associated with this paper can be directed to the lead contact, Tobias Schmidt (tobiasschmidt@ethz.ch).

### Materials availability

This study did not generate new materials.

### Data and code availability

The data displayed in the Figures are available on reasonable request to T.S.S. The underlying project database includes proprietary information that was used under license from Bloomberg (besides many other open data sources) and so is not publicly available. It can be obtained upon reasonable request to T.S.S. with permission of Bloomberg. The Python code used to perform the analysis of this study has been deposited on GitHub (https://github.com/fmueller93/GeothermalLearning2025) and permanently archived on Zenodo (Zenodo: https://doi.org/10.5281/zenodo.21776541) and is publicly available as of the date of publication.

## Acknowledgments

The authors thank all expert interviewees for their valuable time and insights, and Finn Schweden for excellent research assistance. This work received funding from Innosuisse (the Swiss innovation agency) through the AEGIS-CH project (PFFS-21-48/Agreement Nr. 2155009973) and from the Swiss State Secretariat for Education, Research and Innovation (SERI) (contract number: 22.00541) as part of the European Union Horizon Europe Project PRISMA. The opinions expressed and arguments employed herein do not necessarily reflect the official views of Innosuisse, the Swiss Government, or the European Commission.

## Author contributions

Conceptualization: T.S.S., B.S., and F.M.; methodology: F.M., B.S., and T.S.S.; investigation: F.M., B.S., and T.S.S.; visualization: F.M. and B.S.; funding acquisition: B.S. and T.S.S.; supervision: B.S. and T.S.S.; writing—original draft: F.M.; writing—review and editing: T.S.S. and B.S.

## Declaration of interests

The authors declare no competing interests.

## Declaration of generative AI and AI-assisted technologies in the writing process

During the preparation of this work, the authors used Claude Opus 4.6 for language improvements and consistency checks. After using this tool or service, the authors reviewed and edited the content as needed and take full responsibility for the content of the publication.

## STAR★Methods

### Key resources table

**Table:** 

REAGENT or RESOURCE	SOURCE	IDENTIFIER
**Deposited data**		

Geothermal power plant database (514 plants, 206 with cost data)	This study	Available on request to T.S.S.
Bloomberg New Energy Finance (BNEF) geothermal data	Bloomberg New Energy Finance	https://about.bnef.com/
Country update reports from geothermal world/continental congresses	Various	https://worldgeothermal.org/geothermal-data/conference-paper-database
ThinkGeoEnergy portal data	ThinkGeoEnergy ehf.	https://www.thinkgeoenergy.com/
IMF exchange rate data	International Monetary Fund	https://data.imf.org/
Federal Reserve exchange rate data	Federal Reserve System	https://fred.stlouisfed.org/
US Consumer Price Index	U.S. Bureau of Labor Statistics	https://www.bls.gov/cpi/
Analysis code	This study (GitHub)	GitHub: https://github.com/fmueller93/GeothermalLearning2025; Zenodo: https://doi.org/10.5281/zenodo.21776541

**Software and algorithms**		

Excel	Microsoft	https://www.microsoft.com/en-us/microsoft-365/excel
MAXQDA	Verbi Software	https://www.maxqda.com/
Python v3.14	Python Software Foundation	https://www.python.org/
pandas v2.3	PyPI	https://pypi.org/project/pandas/
NumPy v2.4	PyPI	https://pypi.org/project/numpy/; RRID:SCR_008633
openpyxl v3.1	PyPI	https://pypi.org/project/openpyxl/
statsmodels v0.14	PyPI	https://pypi.org/project/statsmodels/; RRID:SCR_016074
SciPy v1.17	PyPI	https://pypi.org/project/scipy/; RRID:SCR_008058
Matplotlib v3.10	PyPI	https://pypi.org/project/matplotlib/; RRID:SCR_008624
GeoPandas v1.1	PyPI	https://pypi.org/project/geopandas/

**Other**		

Interview guide/questionnaire	This study	Table S9
Expert rating framework for technology characteristics	Malhotra and Schmidt^32^; Sievert et al.^33^	https://doi.org/10.1016/J.JOULE.2020.09.004; https://doi.org/10.1016/J.JOULE.2024.02.005

### Method details

#### Quantitative data and analysis

##### Approach and scope

We study geothermal power plants built since 1950 (when the first modern plants came online) and put into operations before 1^st^ January 2025.^35^^,^^70^ To our best knowledge, all global electricity-producing plants that came online since 1950 are included in the data, including later-decommissioned plants. In total, there are 514 plants that are or have been operational. In addition, the database covers 439 plants that are planned, abandoned, or are under construction. 19 of the abandoned projects were drilled to a large part, so that we count them for experience and cumulative capacity (see below).

#### Data sources and verification

Initial data came from multiple sources, most importantly Bloomberg New Energy Finance (BNEF), the most recent country update reports^71^ from the geothermal world or continental congresses, the global portal ThinkGeoEnergy,^72^ and national portals. In the second step, this data was consolidated, and we verified the completeness of the list of plants. Third, we cross-checked and filled missing values, using resources from national and local governments, industry organizations, and operators and suppliers. Furthermore, industry contacts, many of them made during several industry congresses, were used to find resources and verify data. Specific data points, especially outliers and important data gaps were filled during interviews (see below) and through individual requests per mail.

#### Projects considered for the calculation of cumulative capacity

Cumulative capacity includes all projects that came online since 1950. The initial capacity in 1950 is assumed to be at 300 MW and fully located in Italy.^35^^,^^70^ The cumulatively deployed national and global capacity is calculated from all projects until the start of the year in which a plant became operational. Thus, all projects that came online within one year are assigned the same cumulative capacity. Included in the cumulative capacity are all projects that finalized their drilling phase, even if the plant did not become operational. The underlying reasoning is that experience is gained even if a project does not go operational, as the operators gather experience about drilling the local geology and local hydrogeology, which is decisive for project costs. Projects that were abandoned before a significant amount of drilling was done, including projects that did not drill more than a dedicated exploration well, are not included in the cumulative capacity.

#### Converting currencies and deflating

Values are given in USD_2024_. Whenever the input data was presented in another currency, we converted it to USD for the respective year using annual exchange rates from the International Monetary Fund^73^ or the Federal Reserve System.^74^^,^^75^ Subsequently, nominal values were converted to real (2024) USD by applying the US Consumer Price Index.^76^

In the regression models that adjust for exchange rate fluctuations, we introduce a correction factor that normalizes investment costs across different currency areas to a consistent base year exchange rate.^38^

#### Cost data sample

Cost data used for the regression models is available for 206 out of the 514 operational or formerly operational plants (40%). Cost data coverage tends to be higher for more recent projects and is scarce for projects built before 2000 and also varies by country (see Table S2). We do not expect a systematic bias on the cost data from these limitations.

One plant is considered an outlier and manually removed from the dataset (Bad Blumau plant in Austria). This plant is primarily used for heat generation and has less than 1 MW electrical capacity, thus exceeding a specific cost of 100 USD/W.

#### Experience curve model

We use the well-established^29^^,^^33^^,^^38^ formula

$$C_{t}=C_{0}{\left(\frac{x_{t}}{x_{0}}\right)}^{b}\text{,}$$

where C_t_ is the plant cost in year t, C_0_ is the plant cost in year 0, x_t_ is the cumulative capacity in year t, x_0_ is the cumulative capacity in year 0, and b is the rate of cost reduction.^13^

The ER, i.e., the percentage cost reduction from a doubling of cumulative capacity, is then given by

$$ER=1-2^{b}$$

For our empirical estimation of the ER, we can rewrite the first equation in its logarithmic form:

$$\log\left(C_{t}\right)=\alpha +\beta \;\cdot \log\left(x_{t}\right)+\delta D_{t}+\epsilon_{t}\text{,}$$

where α = log(C_0_), β = b, D_t_ is a vector of control variables, and ϵ_t_ is the error term.

#### Regression model

Two model specifications (Models 2 and 4) exclude small projects (with less than 5 MW output), as a skewing of the results was expected due to the increased popularity of such plants (Figure 2A) in recent years and the suspected negative scale effect, i.e., the expectation that these projects would be expensive.^19^ Models 3 and 4 correct for exchange rate fluctuations, which can be suspected to skew the observed ERs due to the long time horizon of observations and many plants in soft-currency countries.^38^ All models use ordinary least squares regressions with heteroscedasticity-robust standard errors.

#### Qualitative data and analysis

##### Expert selection

The expert selection considered three aspects. First, we selected experts with a broad view of the industry and actor network. Second, we aimed at covering all relevant parts of the value chain. Third, we looked for experts to broaden and balance the geographic distribution of experts.

An initial selection of experts was found through industry reports and publications. Experts were approached by the authors at eight industry congresses and five site visits between October 2023 and October 2025, over LinkedIn, and by e-mail.

Experts were contacted on a rolling basis. Due to contacts from the network and direct referrals, the response rate to our requests was high. Only 20 experts that we contacted did not respond to our interview request. We continued recruiting experts until no additional relevant insights were observed.^77^ An anonymized list of interviewees is provided in Table S8.

#### Interview structure

The first interviews were conducted in an explorative way to refine a questionnaire for subsequent in-depth interviews. Based on their expertise, experts were selected either for long interviews covering the entire questionnaire, for short interviews covering only specific aspects of the questionnaire, or for interviews to classify the future technologies EGS and CLGS (see below). Interviewees were sent an interview guide in advance that allowed them to prepare for the interview, see Table S9. The interview guide provided background information on the research project and explained the objectives of the interview. Interviews were held under the Chatham House rule, which states that “participants are free to use the information received, but neither the identity nor the affiliation of the speaker(s) [.] may be revealed.”^78^ In total, 51 interviews were conducted between October 2023 and May 2025. Short interviews took between 10 and 20 min; long interviews took between 50 and 120 min. Each interview was preceded by desk research about the interviewee’s expertise and geothermal plants in which they were involved. Interviews were conducted online or in person, by one or two researchers, in English or German. The discussions were recorded and automatically transcribed. In one case where the interviewee did not consent to recording and for some short interviews which were held at conference venues the interviewers took detailed individual notes.

#### Interview coding

Interview transcripts and notes were analyzed using software for computer-assisted qualitative analysis (MAXQDA 24).^79^ The analysis followed a systematic labeling of each statement. In total, 280 segments were labeled with one or more of 25 different codes and consolidated subsequently.

#### Approach to derive technology characteristic ratings for future technologies

Eleven qualified experts were asked to assess existing and future geothermal technologies regarding technology-inherent characteristics following ref.^28^ The experts were asked to rate hydrothermal, EGS and CLGS in terms of their project design complexity and need for customization on a scale from 1 to 6, which, for both metrics, had other technologies given as references. The ratings were later converted to a range from 1 to 3. For technology characteristics, we introduced one variation from the original framework by Malhotra and Schmidt: Instead of “design complexity”, we use “project design complexity” in this study, to account for the fact that many of the challenges associated with geothermal energy have to do with project management and are not limited to engineering design.

#### Value and validity of expert elicitation to estimate ERs

A promising approach to estimate experience rates for emerging technologies leverages expert elicitations not to predict future costs directly but to assess technology-inherent characteristics that are empirically linked to cost reduction potential. This approach proves more reliable than asking experts directly for cost estimation, a task at which experts performed poorly, especially in nascent technologies.^80^^,^^81^ Meng et al. (2021) demonstrate that model-based projections using experience curves have outperformed expert elicitations in forecasting energy technology costs,^18^ yet constructing experience curves for novel technologies is hampered by limited deployment data. Sievert et al. (2024) address this gap by asking experts to rate the design complexity and customization needs of technology components, which can be judged reliably by experts,^80^ and then mapping these ratings onto empirically grounded experience rate distributions following the technology typology of Malhotra and Schmidt (2020). Crucially, Sievert et al. (2024) validated this method ex-post against observed cost trajectories for concentrated solar power, onshore wind, and solar photovoltaics, with projected cost ranges overlapping 83–100% with actual costs. This suggests that expert-assessed technology characteristics offer a robust basis for deriving experience rates where historical deployment data are insufficient, as is the case for EGS and CLGS.

### Quantification and statistical analysis

All statistical analyses were performed using Python and the packages listed in the key resources table above. The analysis code is available at https://github.com/fmueller93/GeothermalLearning2025.git.

Global and country-level experience rates were estimated using ordinary least squares (OLS) regression on the log-linearized experience curve model. All regression models use heteroscedasticity-robust standard errors (White standard errors). The sample size for the global models is *n* = 206 plants with cost data (or *n* = 173 for models excluding plants below 5 MW). For country-level models, sample sizes vary by country and are reported in Table S4. In each case, n represents the number of individual geothermal power plants.

Confidence intervals (CIs) for the experience rate estimates are reported at the 90% level (Figures 2 and 3; Tables S3 and S4). Statistical significance of the experience rate (i.e., whether the learning parameter b differs from zero) is assessed based on whether the CI excludes zero. Detailed regression parameters, including coefficients, standard errors, *p*-values, and R^2^ values, are reported in Tables S3 (global models) and S4 (country-level models).

For the qualitative analysis, interview data were coded using MAXQDA 24. A total of 280 coded segments were labeled with 25 codes. Descriptive statistics of expert ratings for technology characteristics (project design complexity and need for customization) are reported as means with individual ratings shown in Figures 4D–4F and detailed statistics in Table S6.

One outlier (Bad Blumau plant, Austria) was excluded from the cost dataset based on predefined criteria: the plant is primarily used for heat generation, has less than 1 MW electrical capacity, and exhibits a specific cost exceeding 100 USD/W, which is more than an order of magnitude above other plants.

### Additional resources

Analysis code repository: The Python code used for all quantitative analyses in this study is publicly available at https://github.com/fmueller93/GeothermalLearning2025.git.

## Supplemental information

Document S1. Figures S1, S2, Tables S1–S9, Notes S1, and S2
