# Supplementary material for: Experience rates of current and future geothermal power technologies
Source: iScience. 2026 Aug 12;29(8):117113. doi: 10.1016/j.isci.2026.117113 (PMC13499071; doi:10.1016/j.isci.2026.117113)
Supplement: Supplementary file 1 [file mmc1.pdf]

iScience, Volume *29*

Supplemental information

Experience rates of current and future geothermal power technologies

Florian Mueller, Bjarne Steffen, and Tobias S. Schmidt

Figure S1.

Variation of Fig 1B-M with cumulative capacity instead of annual deployment.

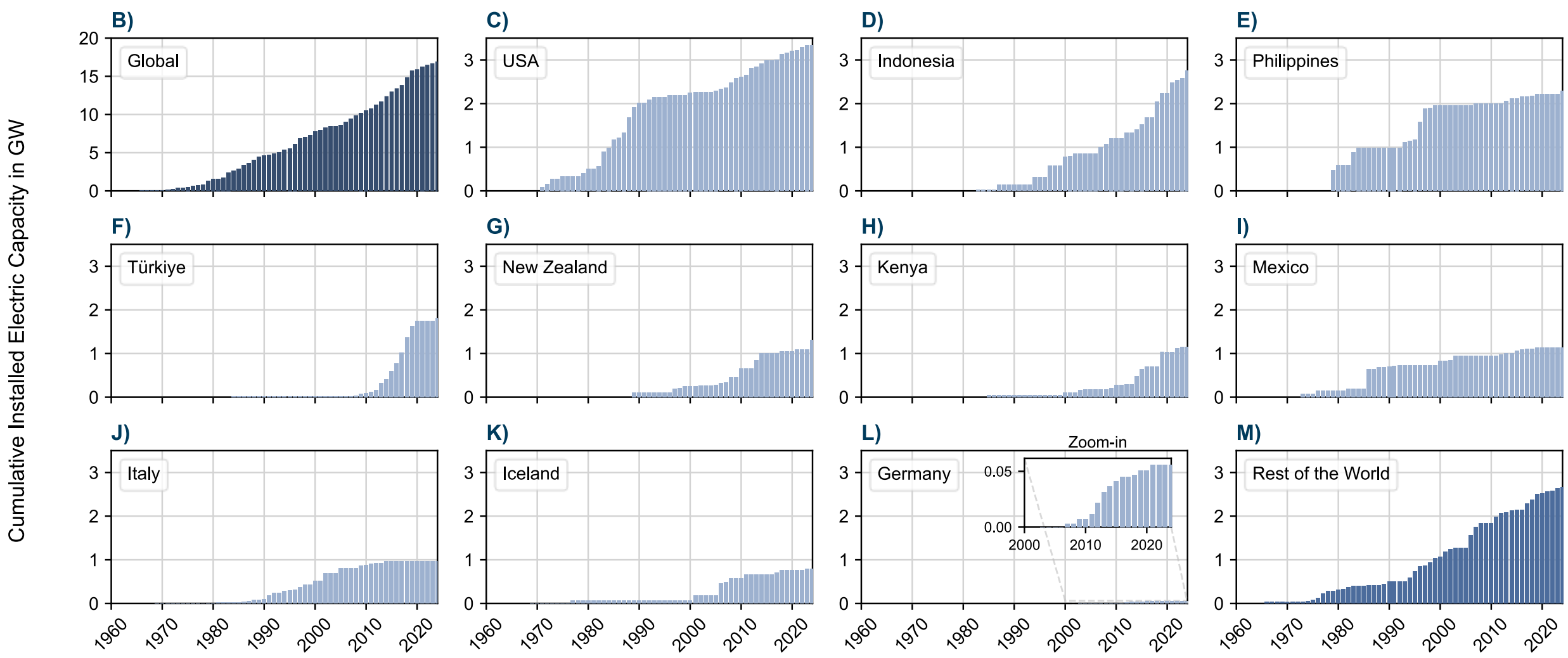

Figure S2.

Resource temperature as a 5-year rolling average of projects with cost data (n = 97) and all projects in the database (n = 269).

Resource temperatures decline in the long-term, driven by declines in individual countries. Fluctuations in the average global temperature are also driven by a varying distribution of projects between nations with different typical temperatures. The uptick in temperatures around 2000 comes from an expansion in Italy's atypically hot fields. In later years, countries with comparably low temperatures, Türkiye and Germany, dominate global new plant installations. After installations in these two countries fell sharply approaching 2020, the global average temperature increased again.

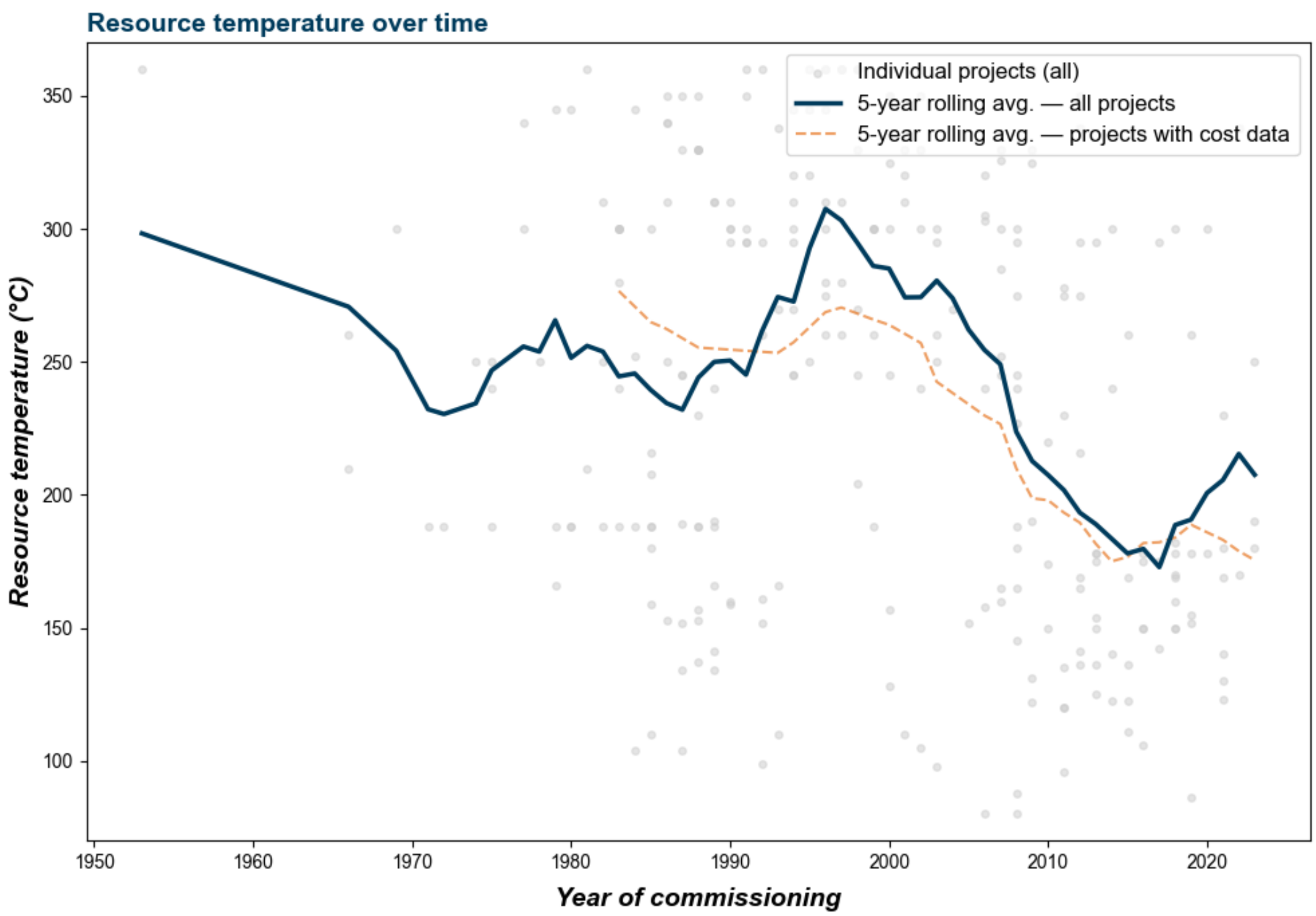

Table S1.

Overview of literature about geothermal cost dynamics and experience rates

Study	Key finding	Method and data set
IEA, 2024[1]	Decline of global installed costs by -80% by 2035	Expert interviews, with the number of experts and their identities not disclosed
Pratama et al., 2025[2]	Global ER -95% / -100% for installed cost	Models use a one-factor experience curve, whereas the second model corrects for scale effects.<br>Data is sourced from IRENA and starts in 2008. It is not entirely clear whether some plant-level data was available or whether the study is based on yearly global averages.
Yao et al., 2021[3]	Global ER -53% / -29% for levelized cost of electricity	The first model uses a one-factor experience curve. The second model uses a multi-factor experience curve, where capacity factor and installation cost are introduced as variables. The dataset is also from IRENA, starting in 2008, and only includes yearly averages, both for cost data and the explanatory variables.
El-Sadi et al., 2024[4]	Local ER for drilling costs: +35%	One-factor experience curve, limited to drilling costs, and from eight wells from two sites and one company, between 2022 and 2024.
Nagy et al., 2013,[5] and Rubin et al., 2015[6]	Local ER of +30% for levelized cost of electricity in the USA	Based a one-factor experience curve and production data from the U.S. Energy Information Administration and cost data from Schilling & Esmundo, which includes 26 data points between 1980 and 2005.[7,8]

Table S2.

Summary statistics related to Fig. 1 and data coverage.

	United States	Indonesia	Philippines	Türkiye	New Zealand	Kenya	Mexico	Italy	Iceland	Germany	Other	**Total**
Projects	118	40	28	74	26	29	31	42	16	15	95	**514**
Avg. capacity (MW)	28.3	64.7	76.3	23.1	54.6	40	36.9	23.1	49.4	3.7	19.1	**33.3**
Projects with cost data	40	24	13	36	16	12	8	2	6	13	36	**206**
Share of projects with cost data	34%	60%	46%	49%	62%	41%	26%	5%	38%	87%	38%	**40%**

Table S3.

Estimates of global experience curve model coefficients related to Fig. 2.

VARIABLES	Model 1	Model 2	Model 3	Model 4
Cumulative deployment	0.267	0.244	0.194	0.255
	(0.140)	(0.122)	(0.277)	(0.286)
Constant	-0.813	-0.711	-0.719	-1.502
	(1.316)	(1.145)	(2.588)	(2.679)
Observations	206	173	206	173
R-squared	0.019	0.021	0.003	0.005
**Experience Rate**	**-20.4%**	**-18.4%**	**-14.4%**	**-19.3%**
**90% Confidence Interval [%]**	[-41.2;-2.6]	[-36.1;-3.0]	[-56.8;16.6]	[-65.4;13.9]
Confidence in sign of ER	97.2%	97.7%	75.8%	81.3%

Robust standard errors in parentheses

Table S4.

Estimates of local experience curve model coefficients related to Fig. 3.

VARIABLES	United States	Indonesia	Philippines	Türkiye	New Zealand	Kenya	Germany
Cumulative deployment per country	-0.281	0.439	0.103	0.130	0.613	-0.0411	-0.0439
	(0.538)	(0.173)	(0.225)	(0.0541)	(0.130)	(0.110)	(0.176)
Constant	3.914	-1.687	0.568	0.647	-2.655	1.779	3.270
	(4.239)	(1.267)	(1.635)	(0.357)	(0.886)	(0.609)	(0.631)
Observations	40	24	13	36	16	12	13
R-squared	0.007	0.163	0.004	0.105	0.544	0.013	0.008
**Experience Rate**	**17.7%**	**-35.5%**	**-7.4%**	**-9.4%**	**-53%**	**2.8%**	**3%**
**90% Confidence Interval [%]**	**[-51.9;55.4]**	**[-65.1;-11.3]**	**[-38.7;16.9]**	**[-16.4;-2.9]**	**[-77.5;-31.8]**	**[-10.2;14.3]**	**[-18.5;20.6]**
Confidence in sign of ER	69.9%	99.4%	67.6%	99.2%	100%	64.5%	59.9%

Robust standard errors in parentheses

Table S5.

Detailed overview of learning factors and quotes, adding further detail to Table 1.

FACTOR	INTERVIEW QUOTE
DIMINISHING PRODUCTIVITY OF NEW SITES	"The sites are more remote than before. […] Just to bring in a turbine [you] might have to rebuild a bridge." (Interview 50)<br>"Today, we build projects in regions that would be way too expensive if we didn't have such high subsidies." (Interview 15)
PROJECT DESIGN COMPLEXITY	"[If] there is too much decline [from the reservoir] your powerplant may not fit your resource [anymore]." (Interview 9)<br>"Designing the heat exchangers and turbine before the well test is risky, but waiting with the design delays the project and also increases costs." (Interview 9)<br>"It is detrimental that there are no stable working relationships between suppliers. The relationships change too often, and good work does not pay off. There are many firms only trying to be cheap […]. Any you say, "never again with this firm". But then they are there again [at the next project]. And you wonder, "how is this possible?". Well, that is because there are barely any people or they [the project owners] just choose whoever is cheapest." (Interview 7)<br>"Past experience has taught us that we need to make good investments in several areas: good surveys before a project starts, for consultants and lawyers, for long-lived casings [i.e., high-grade steel], and for well-qualified and trained rig crews." (Interview 39)<br>"Project owners will take insurance today, even though it is expensive." (Interview 12)
VARYING HYDRO-GEOLOGY	"[You are interested] in permeability and temperature, [but these factors] don't have very good geophysical signatures. […] It's always going to be an interpretation of some sort. […] But, in the end, you have to drill to test your theory […] but drilling is expensive." (Interview 9)<br>"Especially the temperature decline in the reservoir is hard to predict." (Interview 9)<br>Many plants […] have failed or could not fulfill their power purchase agreements [after a reservoir decline and following inefficiencies in the power generation system]." (Interview 9)
TACIT NATURE OF DRILLING KNOWLEDGE	"We brought in experts from [a country with very high experience in geothermal well drilling]. But, we all learnt, these experts had no clue how to drill [the local geological structure] here." (Interview 33)
REGULATORY DENSITY AND VARIANCE	"The subsidy rules are so complicated that we long considered building without subsidy [despite an attractive 40% CAPEX subsidy]. However, fully redeveloped our business model, so it fits the subsidy scheme now." (Interview 7)<br>"Noise regulations have increased drastically. We need to limit noise to 35 dB during drilling now." (Interview 46)<br>"In earlier times, the wells were designed as in the oil and gas industry: with as little material as possible. Today it is the opposite. We need higher-grade materials and greater wall thickness. Partially, this is justified, but the authorities are going too far. The wells are over-designed today." (Interview 39)<br>"[Back then] the produced brine could be flown into the sea, so reinjection wells were not necessary." (Interview 9)<br>"The older line shaft pumps are much easier to operate, especially in high-temperature conditions. The challenge is, you put fifteen liters of oil a day into the resource, it accumulates somewhere, and this is a challenge, especially in Europe." (Interview 31)<br>"Nowadays, it is important to get the public onboard early in the process." (Interview 25)<br>"Sometimes local communities don't want to change […]. [They have little political power] but our geologists will get kidnapped." (Interview 24)
KNOWLEDGE TRANSFER FROM OIL AND GAS INDUSTRY	"There is no good learning ecosystem for geothermal. […] The projects are too small and rare. […] We need a constant inflow of skilled workers from the oil and gas industry. […] And, you need to pay attractive wages." (Interview 7)<br>"[Components like] casings, they used to be available from stock […] they are all sold off because nothing happens in oil and gas [anymore]. This means you need to have the casings produced. The lead times […] have tripled […], it's seven to nine months now." (Interview 45)

Table S6.

Summary statistics of expert ratings of project design complexity and need for customization relating to Figure 4D-F**.** Experts were selected to rate only technologies they had experience in and were given the opportunity to skip technologies if they were not confident about a rating.

In Figure 4D-F, probabilistic noise of max. ±0.1 points is applied to individual ratings in the figure to avoid overlap.

	Project design complexity — Mean	Project design complexity — Variance	Need for customization — Mean	Need for customization — Variance	Sample size
Hydrothermal	2.00	0.11	2.39	0.21	11
EGS	2.47	0.05	2.35	0.07	9
CLGS	2.44	0.11	2.23	0.13	10

Table S7.

Expert quotes about cost reduction potential of future technologies. Detailed factors for the future learning potential of hydrothermal, EGS, and CLGS.

TECH.			FACTOR INFLUENCING FUTURE LEARNING POTENTIAL	INTERVIEW QUOTE
Hydrothermal	EGS	CLGS		
	x	x	High project design complexity and continued challenges from varying hydrogeology	"[EGS and CLGS], it's gonna have a lot more […] complexity than they [the start-ups] like to admit. They make it [the complex drilling] look easy. But geology is gonna get in the way of that [and limit scalability]." (Interview 47)<br>"[CLGS] is all about drilling wells that last [long]; that's not easy unless you've drilled a well in the right way. […] Oil and gas wells are not designed for such long lifetimes." (Interview 9)
	x	x	Slightly smaller but still high challenges from varying hydrogeology	"The need for customization is what has been holding back hydrothermal development. […] The new technologies are promising. If geothermal works, then with these [new technologies]." (Interview 9)<br>"The startups have recently demonstrated impressive drilling results. [However,] they [already] have one of the best-explored geologies. All they are learning there will not be immediately transferrable to the next project, because it will be geologically different. […] Economic viability remains a challenge." (Interview 7)<br>"There is no second Marcellus Shale [where unconventional oil and gas extraction was pioneered]. Such a largely uniform geological area does not exist for geothermal applications. […] Geothermal will not learn as fast as unconventional oil and gas extraction did, even though the technologies are similar." (Interview 47)
	x		Increased project design complexity	"Any sort of complicated fracturing geometry doesn't fit [existing reservoir] models, and it's hard to model what temperature decline [is] gonna be." (Interview 9)<br>"If they start to see […] thermal decline, they're going to have go in and do some mitigation […]. You know, cement off thermal fast paths, drill new wells, or refrack and create new pathways […]. Closed-loop will not have these problems […]." (Interview 9)
		x	High initial costs as a challenge	"CLGS is all about drilling costs. It will be challenging to improve drilling speeds and costs fast enough." (Interview 39)
		x	Decreased project design complexity	"CLGS projects are much higher in investment […]. But, they barely have any operating costs. […] I don't need personnel on site, I don't need pumps, I don't need to exchange pumps. So, over the lifetime, both systems [hydrothermal and CLGS] have similar financial KPIs. But, the big difference is, in CLGS I don't have a geological risk." (Interview 15)<br>"Different to hydrothermal and EGS, reservoir cooling is well-predictable in CLGS. […] you can design the power plants for exactly the amount of power that you want" (Interview 9)
x	x	x	Spatial limitation of technologies and regionally differing preferences	"Hydrothermal, EGS, and CLGS are all interesting, but each in different regions." (Interview 15)<br>"Fracking will never be allowed in Europe; forget about it" (Interview 39)<br>"Renewable heat from geothermal is a big topic in Europe, but not competitive in the USA due to low natural gas prices there." (Interview 40)<br>"Iceland and New Zealand; they only talk about natural systems 'cause don't really ever have to do EGS. [In other areas] with […] very high heat gradients […] just injecting water into a well might be enough to create the permeability you need." (Interview 9)
x	x	x	Reduced project risks from mutual compatibility of technologies	"[…] you can use EGS to reduce dry hole risk or make dry holes valuable." (Interview 9)

Table S8.

List of interviewees.

No.	Regional core expertise	Company type	Function/Role (job titles partly slightly reformulated to ensure anonymization)	Short interview	Long interview	Future tech. rating
1	Central Europe	Research	Project Manager		x	
2	North America	Research	Research Scientist		x	
3	Global	Research	Research Engineer	x		
4	Global	Industry	Power Plant Engineer		x	
5	Europe, Middle East, Africa	Drilling rig owner	Commercial Manager	x		
6	Europe, Mid. East, Latin America	Start-up	Head of Business Development		x	
7	Central and Northern Europe	Global project company	Business Developer		x	x
8	Global	Power plant supplier	Project Engineer	x		
9	North America	Start-up	Resource Development Specialist		x	x
10	Global	Power plant supplier	Commercial Director	x		
11	Global	Project consulting	CEO		x	x
12	Central Europe	Insurance	Insurance Broker		x	
13	Germany	Project operator	CEO		x	
14	Germany	Project operator	Project Manager		x	
15	Europe	Global project company	Business Development		x	
16	Northern Europe	Global project company	Director of External Affairs	x		
17	North America	Oil & gas major	Exploration Consultant	x		
18	Europe, Africa, Latin America	Consulting	Partner	x		
19	North America	Start-up	COO		x	x
20	Europe, Middle East, Africa	Project consulting	Project Engineer		x	
21	Central Europe	Industry association	Executive Director	x		
22	Global	Start-up	Executive	x		
23	Germany	Industry	Construction Project Manager		x	
24	Indonesia	Local project company	Resource Specialist		x	
25	Germany	Local project company	CEO		x	
26	Central Europe	Drilling rig owner	Drilling Operator	x		
27	Global	Supplier	Technology Specialist	x		
28	Global	Management consulting	Partner	x		
29	Switzerland	Local project company	CEO		x	x
30	Central Europe	National government	Technology Specialist		x	x
31	Global	Global project company	Technology Director		x	x
32	Africa	Start-up	CEO	x		
33	Europe, Americas	Knowledge hub	CEO		x	
34	North America	Start-up	Technology Director	x		
35	Oceania and Southeast Asia	Research	Head of Institute	x		
36	Europe, Middle East, Africa	Drilling rig owner	Commercial Manager	x		
37	Global	Global project company	Head of Business Development		x	x
38	Americas, Africa	Think tank	Program Director		x	
39	Europe, Latin America	Drilling consulting	Drilling Engineer		x	x
40	Global	Research	Research Group Leader		x	
41	Central Europe	Knowledge hub	CEO		x	
42	Europe, Middle East, Africa	Project consulting	Engineering Director		x	
43	North America	Research	Technology Specialist	x		
44	Global	Global service firm	Technical Advisor	x		
45	Europe, Middle East	Drilling rig owner	CEO		x	
46	Europe, Latin America	Drilling rig manufacturer	Business Developer		x	
47	Central Europe	Local project company	Project Manager		x	x
48	Global	Research	Professor	x		
49	East Asia	National government	Director	x		
50	Southeast Asia, Americas	Local project company	Head of Technology		x	x
51	Global	Energy company	Program Director	x		

Table S9.

List of guiding questions for the expert interviews.

Q1	What roles have you had in the geothermal or related industries?
Q2	Which geothermal technologies and sub-technologies are you comfortable talking about?
Q3	Which skills/knowledge/capabilities[1] are key to building successful geothermal projects?
Q4	Can you name examples of "learning" in the industry that you have experienced?
Q5	Which skills are key to making enhanced geothermal systems successful?
Q6	Which skills are key to making closed-loop geothermal systems successful?
Q7	What drives complexity in geothermal energy projects?
Q8	Please rate conventional (hydrothermal), enhanced and advanced geothermal energy according to their project complexity on a scale from 1-6.
Q9	Where do you see potential to decrease the complexity of geothermal projects?
Q10	What drives need for customization in geothermal energy projects?
Q11	Please rate conventional (hydrothermal), enhanced and advanced geothermal energy according to their need for customization on a scale from 1-6.
Q12	Where do you see potential to decrease the need for customization of geothermal projects?
Q13	Can some of the previously discussed skills be taken from other industries, if yes, which ones?
Q14	Which of the discussed skills are globally transferrable?
Q15	Which of the discussed skills are specific to local circumstances?
Q16	Can you recommend other interviewees?
Q17	Do you have comments or remarks on this interview guideline?

1) Terms are used interchangeably

Supplementary Note 1 — Drivers of geothermal deployment in different countries

Here, we explain drivers of deployment in selected countries, as seen in Figure 1, that are not covered in the main text. Italy has a long-standing history of geothermal energy use in one field. However, there are no new fields and no strong recent deployment.[13] Germany has only been using geothermal power after 2000, due to the lack of volcanic resources and the comparable higher cost and technological effort to generate power. The deployment has been boosted by strong subsidies, similar to the ones for solar PV and wind at the same time. Nevertheless, the subsidy rules tightened after 2010, where many projects had failed as we find. The subsidies for geothermal power were phased out entirely after 2022, and large new deployments are not expected.[14] Lastly, Australia stands out as over 1 bn USD were invested in geothermal power projects, nevertheless, none of the projects is generating electricity today.[15]

Supplementary Note 2 — Drilling requirements of closed-loop projects

The currently most advanced closed-loop plant is used as an example for drilled distance. It is the plant by the company Eavor in Geretsried, Southern Germany. The project has a projected power output of 8.2 MW at 349 km (23 kW/km) drilled length. The drilled length is from four loops, each with two 4 km vertical sections and 24 laterals with 3.3 km each, connected to form 12 loops.[16] Nearby hydrothermal projects in comparison have 4 MW power output and 12 km drilled length (310 kW/km) from two wells.[17] Thus, the power output per drilled length is more than 10 times higher in the hydrothermal plant.

Supplementary References

1. International Energy Agency. *The Future of Geothermal Energy - Analysis and Key Findings*. https://www.iea.org/reports/the-future-of-geothermal-energy (2024).
2. Pratama, Y. W. *et al*. Learning, economies of scale, and knowledge gap effects on power generation technology cost improvements. *iScience* **28**, 111644 (2025).
3. Yao, Y., Xu, J. H. & Sun, D. Q. Untangling global levelised cost of electricity based on multi-factor learning curve for renewable energy: Wind, solar, geothermal, hydropower and bioenergy. *J. Clean. Prod.* **285**, 124827 (2021).
4. El-Sadi, K., Gierke, B., Howard, E. & Gradl, C. Review of Drilling Performance in a Horizontal EGS Development. in *Proceedings, 49th Workshop on Geothermal Reservoir Engineering* (2024).
5. Nagy, B., Farmer, J. D., Bui, Q. M. & Trancik, J. E. Statistical Basis for Predicting Technological Progress. *PLOS ONE* **8**, e52669 (2013).
6. Rubin, E. S., Azevedo, M. L., Jaramillo, P. & Yeh, S. A review of learning rates for electricity supply technologies. *Energy Policy* **86**, 198–218 (2015).
7. Schilling, M. A. & Esmundo, M. Technology S-curves in renewable energy alternatives: Analysis and implications for industry and government. *Energy Policy* **37**, 1767–1781 (2009).
8. Homepage - U.S. Energy Information Administration (EIA). https://www.eia.gov/.
9. Junginger, M., Sark, W. V. & Faaij, A. *Technological Learning in the Energy Sector: Lessons for Policy, Industry and Science*. (2010).
10. Sanyal, S. K. & Morrow, J. W. Success and the learning curve effect in geothermal well drilling - A worldwide survey. in *Thirty-Seventh Workshop on Geothermal Reservoir Engineering* (2012).

11. Ferioli, F., Schoots, K. & van der Zwaan, B. C. C. Use and limitations of learning curves for energy technology policy: A component-learning hypothesis. *Energy Policy* **37**, 2525–2535 (2009).

12. Yeh, S. & Rubin, E. S. A review of uncertainties in technology experience curves. *Energy Econ.* **34**, 762–771 (2012).

13. Vaccaro, M. *et al.* Geothermal Energy Use, Country Update for Italy. in *Proceedings, European Geothermal Congress 2022* (2022).

14. Weber, J., Born, H., Pester, S., Schifflechner, C. & Moeck, I. Geothermal Energy Use in Germany, Country Update 2019-2021.

15. Beardsmore, G., Ballesteros, M., Davidson, C., Larking, A. & Pujol, M. Country Update-Australia. in *Proceedings World Geothermal Congress 2023* (2023).

16. Eavor Technologies Inc. What The Experts Say Archives. *Eavor Blog* https://eavor.com/category/what-the-experts-say/ (2025).

17. Informationsportal Tiefe Geothermie. Holzkirchen | Informationsportal Tiefe Geothermie. https://www.tiefegeothermie.de/projekte/holzkirchen (2025).
